# Raman spectroscopy detects metabolic signatures of radiation response and hypoxic fluctuations in non-small cell lung cancer

**DOI:** 10.1186/s12885-019-5686-1

**Published:** 2019-05-20

**Authors:** Samantha J. Van Nest, Leah M. Nicholson, Nils Pavey, Mathew N. Hindi, Alexandre G. Brolo, Andrew Jirasek, Julian J. Lum

**Affiliations:** 1 Department of Physics and Astronomy, University of Victoria, PO BOX 1700 STN CSC, Victoria, BC V8W 2Y2 Canada; 2 Trev and Joyce Deeley Research Centre, BC Cancer, 2410 Lee Avenue, Victoria, BC V8R 6V5 Canada; 3 Department of Chemistry, University of Victoria, PO BOX 3065, Victoria, BC V8W 3V6 Canada; 4 Department of Physics, I.K. Barber School of Arts and Sciences, University of British Columbia-Okanagan, 3187 University Way, Kelowna, BC V1V 1V7 Canada; 5 Department of Biochemistry and Microbiology, University of Victoria, PO BOX 1700 STN CSC, Victoria, BC V8W 2Y2 Canada

**Keywords:** Raman spectroscopy, Non-small cell lung cancer, Hypoxia, Reoxygenation, Ionizing radiation, Immunofluorescence

## Abstract

**Background:**

Radiation therapy is a standard form of treating non-small cell lung cancer, however, local recurrence is a major issue with this type of treatment. A better understanding of the metabolic response to radiation therapy may provide insight into improved approaches for local tumour control. Cyclic hypoxia is a well-established determinant that influences radiation response, though its impact on other metabolic pathways that control radiosensitivity remains unclear.

**Methods:**

We used an established Raman spectroscopic (RS) technique in combination with immunofluorescence staining to measure radiation-induced metabolic responses in human non-small cell lung cancer (NSCLC) tumour xenografts. Tumours were established in NOD.CB17-Prkdc^scid^/J mice, and were exposed to radiation doses of 15 Gy or left untreated. Tumours were harvested at 2 h, 1, 3 and 10 days post irradiation.

**Results:**

We report that xenografted NSCLC tumours demonstrate rapid and stable metabolic changes, following exposure to 15 Gy radiation doses, which can be measured by RS and are dictated by the extent of local tissue oxygenation. In particular, fluctuations in tissue glycogen content were observed as early as 2 h and as late as 10 days post irradiation. Metabolically, this signature was correlated to the extent of tumour regression. Immunofluorescence staining for γ–H2AX, pimonidazole and carbonic anhydrase IX (CAIX) correlated with RS-identified metabolic changes in hypoxia and reoxygenation following radiation exposure.

**Conclusion:**

Our results indicate that RS can identify sequential changes in hypoxia and tumour reoxygenation in NSCLC, that play crucial roles in radiosensitivity.

**Electronic supplementary material:**

The online version of this article (10.1186/s12885-019-5686-1) contains supplementary material, which is available to authorized users.

## Background

Radiation therapy (RT) plays a vital role in the management of non-small cell lung cancer (NSCLC), where definitive and concurrent RT is a standard treatment approach [1–7]. Meta-analysis of RT techniques for the treatment of medically inoperable early-stage NSCLC reported a 2-year overall survival estimate of 53% for conventional (60 Gy total, 2 Gy fractions) RT [8]. Improvements to radiation dose delivery and hypofractionation using stereotactic body radiation therapy (SBRT) have resulted in an improved 2-year survival of 70% [8]. Despite these advances, local failure at 5 years following SBRT is reported as high as 20% [9]. This can be attributed in part to patient-specific heterogeneity both at the genomic and metabolic level of the tumour environment that leads to radioresistance and metastasis [1]. Better strategies for local control would mitigate these potential issues and improve clinical management in this setting. This includes consideration for biology-based treatment strategies [9] and individualized dose escalation [10] to address patient-specific heterogeneities.

Both intrinsic and extrinsic factors can influence tumour response and overall outcome of RT [11]. Hypoxia is a prominent feature in NSCLC and a well described characteristic of radioresistant tumours [12–14]. Biologically, hypoxia exerts pleiotropic effects on tumour cell biology with a key role of activating hypoxia inducible factor 1 (HIF-1), a transcriptional factor responsible for regulating cell survival, metabolism and invasiveness [12, 15, 16]. However, hypoxia is not a static event and cycles of hypoxia in different spatial regions of the tumour and throughout treatment can influence radiosensitivity, particularly when RT is given as fractionated doses [16, 17]. As a result of this regional distribution of oxygen, RT is cytotoxic to aerobically active cells. In contrast anaerobically active cells endure less radiation damage and can survive in order to resume growth and proliferation following reoxygenation [18]. However, the precise in vivo metabolic changes that occur in these micro-tumour niches after radiation treatment is unclear. Such information may be important to tailor radiosensitization strategies that target specific alterations within a given tumour.

Non-invasive techniques to measure transient changes in tumour hypoxia include electron paramagnetic resonance oximetry [19], magnetic resonance imaging [20] and photoacoustic imaging [21]. Investigation of tumour hypoxia for prognostic value in RT has also been extensively investigated [22–25]. However, additional factors affecting RT response beyond hypoxia may also warrant consideration. Nucleic acid break repair markers [26], circulating cell-free DNA [27] and genetic host factors (e.g. single-nucleotide polymorphisms) [28, 29] have also been investigated as predictors of radiation response. Unfortunately, these techniques only assess a single indicator of the radiation response (e.g. hypoxia or DNA break repair). As such, they have not been implemented to monitor radiation resistance clinically, presumably due to limitations including insufficient clinical validation, invasiveness and cost.

Raman spectroscopy (RS) is a non-destructive and label-free optical spectroscopic technique with the potential for in vivo tracking of overall biomolecular changes in cells and tissue. These characteristics are favorable for biomedical applications and, in particular, allow for non-targeted monitoring of radiation resistance indicators in cells and tissues. Raman spectroscopy has shown promise as a way to monitor radiation response in a variety of systems including human lens epithelium [30], blood lymphocytes [31], oral [32], cervical [33], breast, prostate and lung cancers [34–37]. In particular, applications of RS to NSCLC have revealed distinct radiation-induced metabolic alterations in cells [35, 38] and tumour xenograft tissue [36]. These responses manifest as an accumulation of glycogen in irradiated samples when studied 72 h post treatment. The observed glycogen accumulation was found to be associated with increased GSK-3β and AMPKα signaling in vitro, however the dynamics within the tumour can be different and have not been investigated.

In this study, we investigate the nature of the radiation-induced metabolic alterations observed in NSCLC tumour xenografts using RS. In particular, the extent of glycogen accumulation in tumour cells differed as a result of radiation treatment. For example, there were detectable but lower levels of glycogen in irradiated samples after acute irradiation compared to unirradiated controls. This reduction of glycogen recovered and peaked on day 3 in irradiated tumour and could be observed even up to 10 days post treatment. Moreover, perturbations in the Raman glycogen signature are correlated to tumour regression and could be linked to tumours that were undergoing reoxygenation. Taken together, RS is a potentially valuable technique that links hypoxia, glycogen and reoxygenation as key factors in radiation responses of NSCLC.

## Methods

### Cell lines and tumour implantation

All animal procedures were approved by the University of Victoria Animal Care Committee and performed in accordance with the guidelines set forth by the Canadian Council on Animal Care. 6–8 week old NOD.CB17-Prkdc^scid^/J female mice were obtained from BC Cancer Research Center Animal Resource Center Vancouver (colony originally sourced from The Jackson Laboratory, Bar Harbor, ME, USA). Animals were housed in HEPA filter microisolater cages within a bioexclusion containment room and allowed access to food and water ad libitum. Animals were acclimatized for 1 week prior to study initiation.

The human non-small cell lung adenocarcinoma cell line H460 (purchased from American Type Culture Collection, Manassas, VA, USA, ATCC# HTB-177, passage# 3, not listed as misidentified, tested pathogen-free with Charles River Laboratories, Montreal, QC, Canada) was used in this study. Cells were cultured according to previously established protocols [35] and subsequently injected subcutaneously in the right flank of each mouse at a concentration of 5 × 10^6^ cells in 0.1 ml phosphate buffer saline (PBS). Subcutaneous tumour models were selected due to practical and technical considerations involving the H460 cell line.

### Tumour irradiation and harvesting

Pre-determined exclusion criteria disqualified animals from receiving radiation treatment and continuing in the study if the tumour volume was below 80 mm^2^ or above 130 mm^2^. Tumours were measured three times per week with digital calipers and animals were randomized into treatment groups once their tumour size reached the pre-determined endpoint. On average, tumour volume was 97 +/− 7 mm^2^, and grew with a tumour volume doubling time of 6.7 +/− 0.6 days. Investigators were blinded to radiation dose delivered to a given mouse.

Mice were treated using a small animal radiation research platform (SARRP, Xstrahl, Gulmay Medical Inc., Suwanee, GA), according to previously described techniques [37]. Briefly, mice were anaesthetized using isoflurane inhalation (2%, in oxygen) and imaged using a single cone beam computed tomography (CBCT) scan. Treatment plans were made using Muriplan (Gulmay Medical Inc.) and single fractions of 0 or 15 Gy were delivered to the tumour (parallel irradiation studies) using two 220 kVp parallel opposed fields, collimated to 1 × 1 cm^2^, at a dose rate of 4 Gy/min [39]. For all studies involving evaluation of carbonic anhydrase IX (CAIX) expression, tumours were irradiated with a clinical linear accelerator using previously established protocols [36].

Tumours were measured and pimonidazole (PIMO) was injected intraperitoneally at 100 mg/kg one hour prior to euthanasia [40]. Tumours used for CAIX studies were not injected with pimonidazole prior to euthanasia. At 2 h, 1 day, 3 days or 10 days post irradiation mice were euthanized through isofluorane overdose (5%, in oxygen) and cervical dislocation. Immediately following euthanasia, tumours were extracted, embedded in mounting medium (Tissue-Tek O.C.T. Sakura Tinetek Europe B.V., The Netherlands), flash frozen in liquid nitrogen and stored at − 80 **°**C.

### Ex vivo Raman microscopy

Three consecutive tissue sections (each with a thickness of 20 μm) were extracted from a given tumour, using a rotary microtome (HM 550; MICROM International GmbH, Walldorf, Germany) and placed on magnesium fluoride slides. A Renishaw inVia Raman Microscope (Renishaw Inc., Illinois, IL, USA) coupled to a 785 nm diode laser (Renishaw) and dry objective (100x, NA = 0.9, Leica Microsystems, Wetzlar, Germany) was used to collect Raman map spectra. Spectra were collected using a thermoelectrically cooled charge coupled device (CCD) detector (Andor Technology, Connecticut, USA). Spectra were acquired for 20 s per point, covering a spectral range of 420–1800 cm^− 1^. Raman maps were collected over an area of either 100 × 100 μm^2^ or 200 × 200 μm^2^ and were acquired using a step size of 15 μm in both dimensions. Two maps were collected from randomly selected regions within each tissue section, leading to a total of six maps collected per tumour (2 maps per section, 3 sections per tumour). This study included a total of 28 mice (8 mice at 2 h and 10 days post irradiation; 4 per dose group, 6 mice at 1 and 3 days post irradiation; 3 per dose group). Sample size was selected based on preliminary Raman tissue studies carried out in our group [36].

### Spectral processing and analysis

The data set consisted of 14,192 spectra prior to outlier removal and reduced to 13,614 spectra after outliers (e.g. saturated spectra) and spectra contaminated with cosmic rays were visually identified and removed from the data set (2 h 0 Gy; *n* = 2049, 2 h 15 Gy; *n* = 1908, day 1 0 Gy; *n* = 1380, day 1 15 Gy; *n* = 1374, day 3 0 Gy; *n* = 1499, day 3 15 Gy; *n* = 1462, day 10 0 Gy; *n* = 1923, day 10 15 Gy; *n* = 2019).

In-house algorithms developed in Matlab (Mathworks, Natick, MA, USA, version R2014B) estimated and subtracted spectral background using a Savitsky-Golay zero-order filter [41], shifted spectra to account for instrument drift using the phenylalanine peak located at 1003 cm^− 1^ and normalized spectra to the total amount of biological material in the sampling volume, as described previously [42]. Principal component analysis (PCA, standard function available in Matlab) was used to separate spectral features that contribute variability in the data set due to radiation exposure and those contributed from other sources. Due to the overlapping nature of major Raman bands arising from biomolecular components in tissue, only biomolecules with several contributing spectral bands arising on the same side (positive or negative) of the principal component (PC) axis are definitively assigned and further considered in our analysis. Furthermore, biomolecular changes identified through RS are discussed in terms of relative changes as outlined in the positive and negative features of a given PC, rather than absolute changes in specific biomolecules.

### Immunofluorescence imaging

Tissue was sectioned to 10 μm using a rotary microtome, placed on glass slides and fixed with PBS containing 2% paraformaldehyde (Alfa Aesar, MA, USA) for 1 min. Fixed tissue sections were washed twice with PBS then blocked using PBS with triton-x (0.1%, Fisher Scientific) and Bovine Serum Albumin Fraction V (BSA, 2%, Roche Diagnostics, Mannheim, De). Cells were incubated at room temperature for one hour with anti-CAIX (1:50 dilution, antibody validated previously [43]) or anti-pimonidazole 1:10 dilution, hypoxyprobe 4.3.11.31 mouse MAb, HPI Inc., MA, USA) antibodies. Sections were then washed twice with PBS followed by a single wash of PBS with Triton-x (0.1%) and BSA (0.5%).

In the case of the singly stained CAIX sections, chicken anti-mouse Alexa Fluor 488 (1:100 dilution, Invitrogen Molecular Probes, OR, USA) was incubated for 30 min at room temperature, after which sections were washed twice with PBS, fixed using 2% paraformaldehyde for 1 min then washed with PBS.

In the case of the doubly stained PIMO and phospho-histone H2AX (γ-H2AX) sections, chicken anti-mouse Alexa Fluor 488 secondary antibody (1:100 dilution, Life technologies, OR, USA) was incubated for 30 min, washed twice with PBS then incubated for one hour with anti-γ-H2AX (1:50 dilution, rabbit MAb, Cell Signaling Technology, MA, USA). This was followed by two washes with PBS and a single wash of PBS with Triton-x (0.1%) and BSA (0.5%). Sections were incubated for 30 min with goat anti-rabbit Alexa Fluor 546 secondary antibody (1:100 dilution, Invitrogen, OR, USA), followed by two washes with PBS, a single wash with 2% paraformaldehyde for 1 min and a final wash with PBS. For both the single and double stained sections nuclear localization was carried out using Vectashield Mounting Medium with DAPI (Vector Laboratories Inc., CA, USA).

Immunofluorescence (IF) images were collected at 20x magnification using an Olympus microscope coupled with a Nuance multispectral imaging system (Perkin Elmer, MA, USA) and multi-spectral laser (Excelitas Technologies). Percent coverage of IF images was assessed using FIJI (ImageJ). An intensity threshold was assigned to grey scale images of a given fluorophore to eliminate non-specific background staining. The percent area of the field of view containing intensities above this threshold was assessed and reiterated for each field of view.

### Co-registration of Raman and immunofluorescence data

Raman mapping and CAIX IF imaging was carried out for the same region of a 20 μm thick tissue section for several tumours irradiated to 0 or 15 Gy. The Raman spectral map was collected using the Renishaw Raman system with a 20 μm step size in each dimension, over a 500 μm × 600 μm region using a 100x Leica dry objective. A white light image of the same region was also collected using the Renishaw system and a 10x Leica dry objective. The CAIX immunofluorescence staining was achieved using the same protocol identified above, with images of DAPI and CAIX collected using 10x magnification.

Spectra contained within the Raman map were analyzed using PCA and the principal component score associated with glycogen (PC1) was plotted as a function of position. Image alignment and cropping was carried out in FIJI. The glycogen spectral map was aligned to the white light image using the associated coordinates with these two images. Next, the CAIX IF image was aligned manually using structural features of the tissue section. The CAIX IF image was then cropped in FIJI using the glycogen map as a mask. In house algorithms were developed in Matlab to segment the IF image and glycogen map into co-registered pixels. Mean IF intensity within a pixel was then compared to glycogen score.

### Statistical procedures

Statistical significance of PC score shifts were tested using a two-sided Wilcoxon rank sum test to a 5% significance level. This test assumes similar standard deviation among the test populations, a condition that is satisfied by the data sets examined. Statistical significance of shifts in mean IF staining was tested using a two-tailed Student’s t-test. Linear fitness of trends were assessed by determining the correlation coefficient between the dataset and a first-order polynomial fit to the dataset using standard Matlab algorithms. Data points outside the 75% prediction intervals for the data set were considered outliers in analysis of CAIX IF intensity vs principal component score.

## Results

### Raman spectroscopy identifies radiation-induced glycogen perturbations as early as 2 h and up to 10 days post irradiation

Non-small cell lung cancer cells were injected subcutaneously in mice and tumours grew to approximately 10 mm in diameter. Tumours received conformal radiation therapy to a single dose of 15 Gy or were untreated, then harvested at 2 h, 1, 3 or 10 days following irradiation. Raman spectra were collected randomly from each tumour and exhibited variation in Raman band intensity based on dose and time of harvest. Mean Raman spectrum +/− the standard deviation for all spectra collected in the unirradiated and irradiated groups at 2 h and 3 days are shown in Additional file 1: Figure S1. To better delineate the in vivo metabolic changes induced following radiation, a combination of RS and PCA was used. Correlated Raman spectral features within dose groups were identified at 2 h, 1, 3 and 10 days post irradiation. In this study, 75% of the total variance in the data set can be described by considering the first five principal components. However, all other components beyond PC1 and PC2 contribute to less than 8% of the total variance in the data set, per component. This is nearly half the variance described by PC2, therefore further analysis focused only on PC1 and 2.

**Fig. 1:**
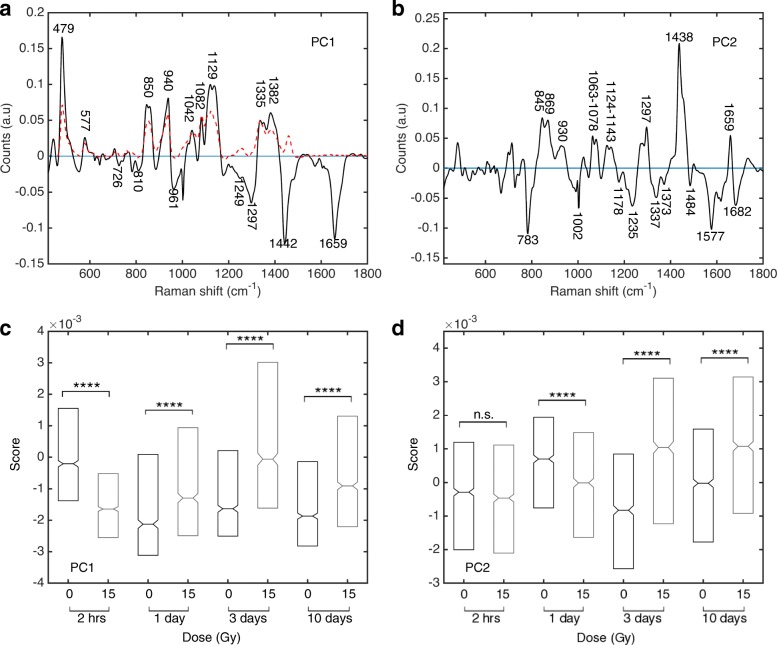
Raman spectral changes linked to glycogen in NSCLC xenograft tissue following irradiation**.** Principal component 1 (**a**) and PC2 (**b**) (black traces) with corresponding box plots of PC1 score (**c**) and PC2 score (d) (Tukey style box plot, outliers left out for clarity, notches indicate 95% confidence interval on the median). Median PC scores are shown for all spectra collected over four tumours (2 h, 10 days) or three tumours (1 day, 3 days) in a single dose group and time point. The dashed red trace in (**a**) represents the Raman spectrum of pure glycogen (sample obtained from Life Technologies Inc., Burlington, ON, Canada). Statistical significance: **** *p* ≤ 0.0001, n.s.- not significant

The first principal component (PC1) isolated in the Raman dataset contributes to 42% of the total variance in the dataset (Fig. 1a). PCs are plotted as intensity (counts, arbitrary units) for each wavenumber in the spectrum contained within this component. The spectral band assignment for PC1 indicated positive spectral features exclusively due to glycogen and negative bands arising from nucleic acids (adenine cytosine, thymine), protein (amide I and III, phenylalanine, carbon vibrational modes) and lipids (carbon vibrational modes; Additional file 1: Table S1). The Raman spectrum of pure glycogen (red trace, Fig. 1a) shows strong overlap with the positive features in PC1, including peaks at 479 cm^− 1^, 577 cm^− 1^, 850 cm^− 1^, 940 cm^− 1^, 1042–1130 cm^− 1^, 1335–1382 cm^− 1^. In addition, previous studies by our group showed that increased PC1 score in the H460 cell line is associated with an increase in glycogen in tumours through Periodic-Acid Schiff staining [36]. This evidence supports the assignment of trends in PC1 to changes in glycogen content in the tissue, as observed through RS.

Similarly, the second principal component (PC2) contributes 14% of the total variance in the dataset (Fig. 1b). Several positive features were due to protein (carbon-nitrogen bands, α-helix associated carbon-carbon vibrational modes, amide I/ III bands) and lipid (carbon-nitrogen, carbon-hydrogen, carbon-carbon bands; Additional file 1: Table S2). In contrast, PC2 revealed a subset of negative features arising due to protein (phenylalanine, tyrosine, amide bands associated with random coil and β-sheet protein structure) and nucleic acids (guanine, adenine). Previous Raman and cell synchronization studies have linked PC2 spectral features to progression through cell cycle [42].

Next, the median PC1 score was determined for the spectra collected from all tumours at each dose and time point studied. The PC1 scores indicate that at 2 h post irradiation there was an initial reduction in glycogen content of irradiated tumours compared to unirradiated tumours (*p* < 0.0001; Fig. 1c). However by 1 day post irradiation, the glycogen levels in irradiated tumours was greater than unirradiated tumours (*p* < 0.0001) along with an overall increase in glycogen levels in irradiated tumours between day 1 and day 3 (*p* < 0.0001) with a corresponding reduction in glycogen levels in the unirradiated tumours from day 1 to 3. By day 10, tumours that received radiation displayed twice as much glycogen compared to unirradiated controls (p < 0.0001) though the total glycogen levels were lower than tumours examined on day 3 post irradiation.

To determine whether these dynamic spectral changes were unique to PC1, a similar analysis of PC2 scores was performed (Fig. 1d). At 2 h post irradiation, the median PC2 scores did not reveal any statistical difference between the irradiated and unirradiated groups (*p* = 0.14). However, there was a positive shift in the PC2 scores 24 h post irradiation, with the unirradiated group shifting more than the irradiated group (*p* < 0.0001). At 3 and 10 days post irradiation, the 15 Gy group shifts to more positive median PC2 score while the unirradiated group shifts to a more negative score compared to day 1. Comparatively, the irradiated tumours displayed at least a 2-fold increase in PC2 score compared to the unirradiated controls at 3 and 10 days (*p* < 0.0001).

### Glycogen expression is correlated to tumour regression in NSCLC

**Fig. 2:**
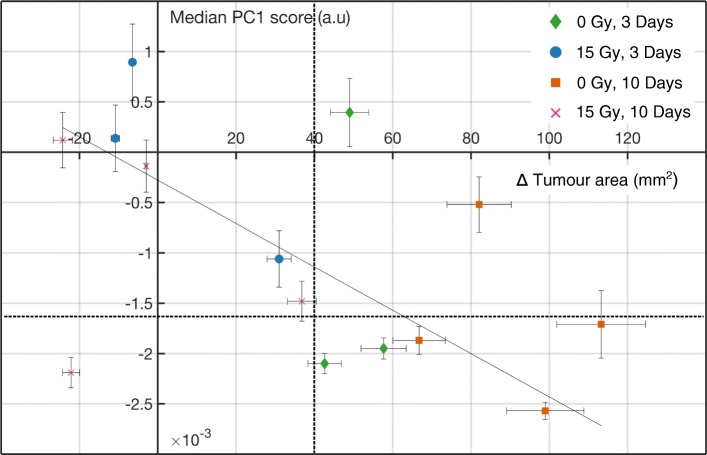
Raman spectroscopy identifies increased glycogen, which is correlated with radiation-induced tumour regression. Median PC1 score as determined using RS is shown as a function of change in tumour area as measured from time of treatment to time of euthanasia. This is plotted for 0 Gy and 15 Gy irradiated tumours harvested at 3 and 10 days. Error bars represent 95% confidence interval on the median PC1 score, and an estimated measurement uncertainty of 10% on tumour area

Given the close relationship between radiosensitivity and the metabolic status of the tumour, we examined whether the response to radiation was associated with variances observed in PC1. As expected, unirradiated tumours increased in size over time (ranging 42 to 113 mm^2^) (Fig. 2). With the exception of two unirradiated tumours, median PC1 scores all fall below − 1.71 × 10^− 3^, indicating minimal glycogen content relative to the remaining tumours in the data set. As such, large unirradiated tumours displayed low glycogen levels. In contrast, irradiated tumours which exhibited partial responses (area increases between 31 and 37 mm^2^) or size reduction (on the order of 3 to 24 mm^2^) at 3 and 10 days post treatment. This resulted in a corresponding and higher median PC1 scores, indicative of increased glycogen. A linear fit to this data set shows that glycogen content (PC1 score) is linearly correlated to change in tumour area (R^2^ = 0.86). We also investigated the link between tumour regression and PC2, and found a weak anti-correlation between the two (R^2^ = -0.55, Additional file 1: Figure S2).

### DNA damage and hypoxia are associated with RT exposure

**Fig. 3:**
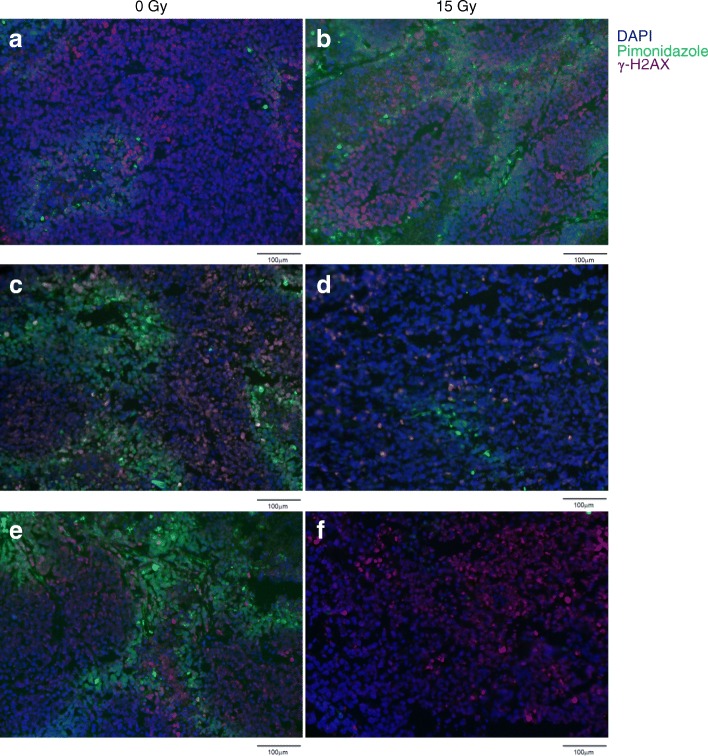
Concurrent immunofluorescence staining of γ-H2AX associated DNA damage, and hypoxia indicated through pimonidazole. Immunofluorescence images of 0 Gy (left panels) and 15 Gy (right panels) tumours harvested at 2 h (**a, b**), 3 (**c, d**) and 10 (**e, f**) days post irradiation. Nuclear localization was made using DAPI (blue), while DNA damage was indicated through γ-H2AX (red) and hypoxia through pimonidazole (green)

**Fig. 4:**
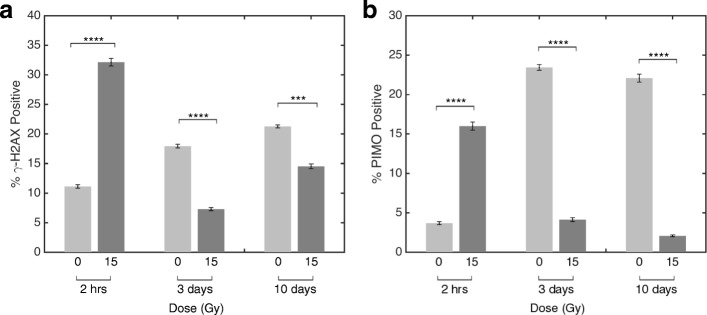
Quantification of immunofluorescence staining of γ-H2AX **(a)** and pimonidazole **(b).** Images were assessed for percent area stained positive for each marker. Mean percent coverage was determined for each dose group and time point (*n* = 18 images per group), with error bars indicating standard error on the mean. **** *p* ≤ 0.0001, *** *p* ≤ 0.001

Given the extent of glycogen changes following RT, we examined whether this is a function of the extent of DNA damage and hypoxia. Pimonidazole stains cells that are exposed to low oxygen conditions [44, 45] while CAIX, a transcriptional target of HIF-1 with a long half-life, was used as a surrogate for cells which may have been previously hypoxic [46]. Furthermore, γ-H2AX staining denoted the localization of radiation-induced DNA double strand breaks [47]. Multi-parametric staining was performed to identify γ-H2AX (red), pimonidazole (green) and DAPI (blue) in irradiated and unirradiated tumours harvested at 2 h, 3 and 10 days post-irradiation (Fig. 3). The average percent area with positive staining was assessed for each dose and time point. As expected, DNA damage was found to be greater in irradiated tumours at 2 h post-irradiation compared to the unirradiated controls (p < 0.0001; Fig. 4a). Similarly, irradiated tumours were found to have increased PIMO staining at 2 h (*p* < 0.0001; Fig. 4b) indicating greater levels of hypoxia at this time.

At 3 and 10 days, unirradiated tumours were found to have increased levels of γ-H2AX compared to irradiated tumours (*p* < 0.0001 day 3, *p* = 0.001 day 10; Fig. 4a). This is likely due to increased levels of hypoxia in unirradiated relative to irradiated tumours at 3 and 10 days as confirmed by PIMO staining (*p* < 0.0001 for both 3 and 10 days; Fig. 4b) and has also been reported in the literature [48].

### Irradiated tumours exhibit higher CAIX expression

**Fig. 5:**
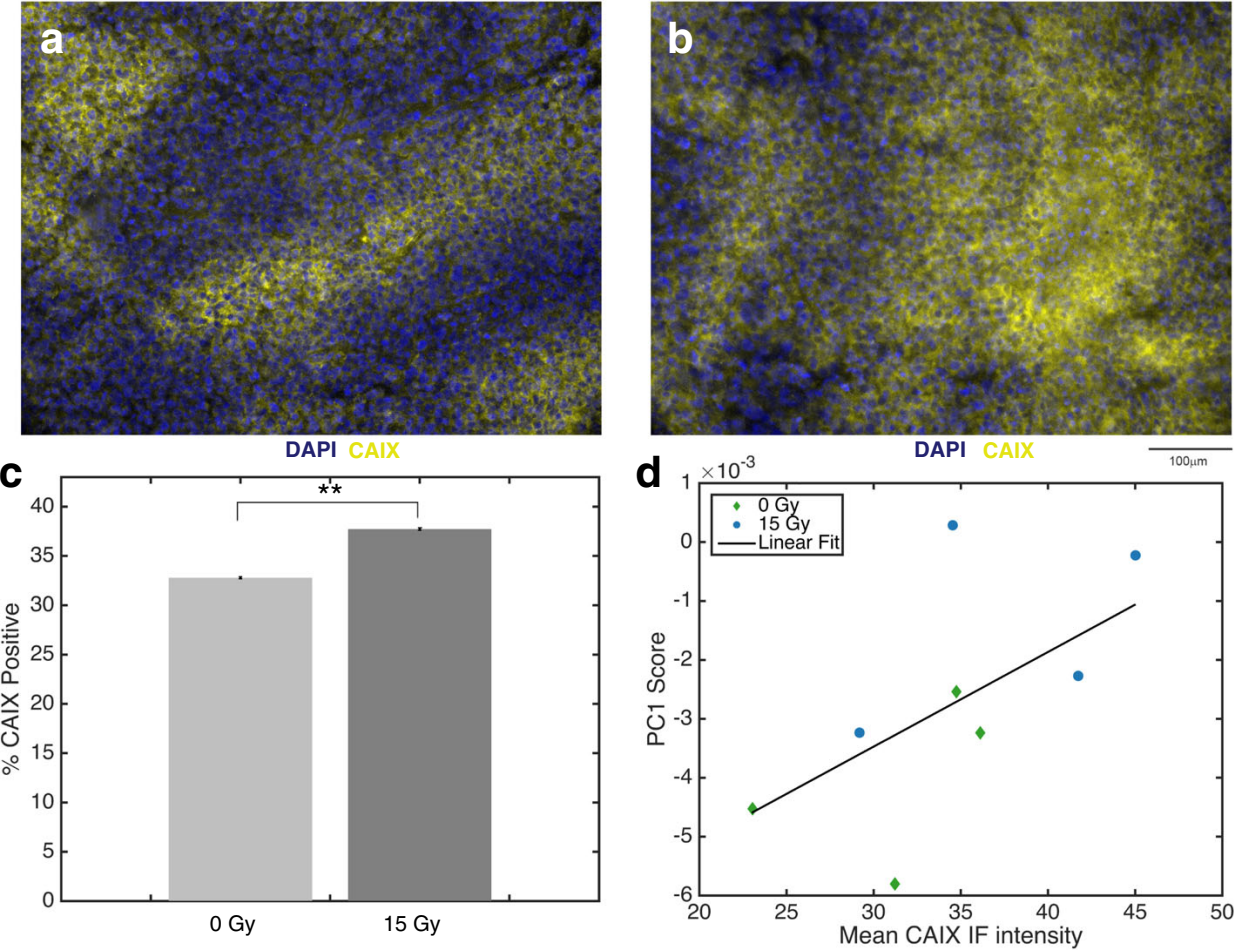
Trends in CAIX expression between irradiated and unirradiated tumours harvested at 3 days post-treatment. Immunofluorescence images showing nuclear localization through DAPI (blue) and surrogate marker for hypoxia through CAIX (yellow) for 0 Gy (**a**) and 15 Gy (**b**) tumours. The mean percent area positive for CAIX over several images per dose group (*n* = 136 for 0 Gy, *n* = 122 for 15 Gy) is shown in (**c**), indicating a statistically significant increase in CAIX staining for irradiated tumours (**p ≤ 0.01). Error bars indicate standard error on the mean. (**d**) Median PC1 score indicating glycogen content is plotted against the mean percent CAIX positive for each 0 Gy and 15 Gy tumour studied at 3 days post-treatment. A linear correlation between glycogen content (PC1 score) and percent CAIX positive for a given tumour is shown (R^2^ = 0.90, *p* = 0.01)

Given the hypoxia results above, the extent of CAIX positive tumour regions was quantified to examine the qualitative degree of tissue oxygenation post irradiation. This analysis revealed positive association between CAIX expression levels (yellow) with tumours irradiated at 0 Gy (Fig. 5a) and 15 Gy (Fig. 5b) and harvested at 3 days post irradiation. In general, irradiated tumours show increased CAIX positive staining compared to unirradiated controls (*p* < 0.01) at 3 days post-treatment. This trend is quantified in Fig. 5c, through the average percent area positively stained for CAIX over all tumours in a dose group.

### Intra-tumour glycogen expression is correlated to CAIX expression

Next, to elucidate the potential impact of CAIX expression on glycogen accumulation tissues collected on day 3 post-treatment were stained with CAIX. Overall, there was a linear relationship (R^2^ = 0.90, *p* = 0.01) between median PC1 score and mean CAIX IF coverage (Fig. 5d). As expected, irradiated tumours fall along the upper right portion of the plot, suggesting increased glycogen levels and increased CAIX coverage while unirradiated tumours tend to lay in the lower left portion, indicating lower glycogen and CAIX levels. PC2 was not significantly correlated to CAIX expression (R^2^ = 0.39, *p* = 0.34, Additional file 1: Figure S3).

**Fig. 6:**
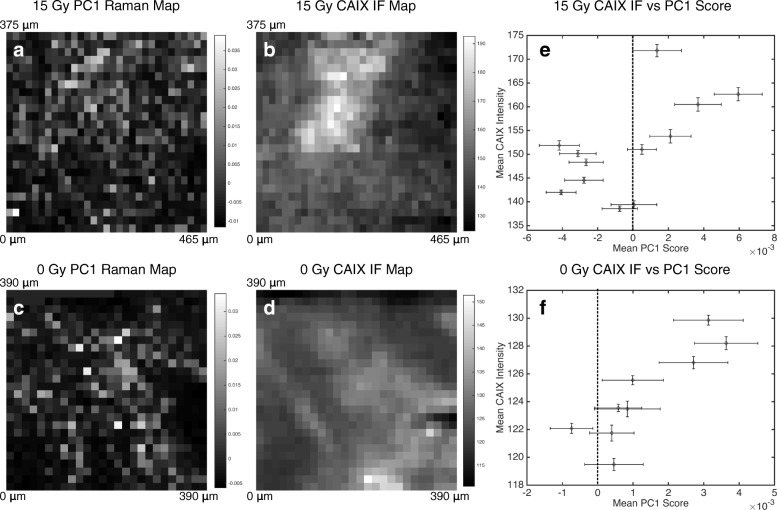
Local correlations between cellular glycogen using RS and CAIX Immunofluorescence. Raman intensity maps of glycogen (PC1), indicating the spatial distribution (20 × 20 μm^2^ pixel size) of glycogen in a 0 Gy (**a**) and 15 Gy (**c**) tissue section (harvested at 3 days). Coincident CAIX immunofluorescence intensity spatial maps (20 × 20 μm^2^ pixel size) for the same region are also shown for 0 Gy (**b**) and 15 Gy (**d**). Bright regions indicate high CAIX stain intensity or high glycogen (PC1 score) at that pixel (**e-f**). The mean CAIX intensity for 100 × 100 μm^2^ regions within each intensity map is plotted against the mean PC1 score in the same region (error bars are the standard deviation), indicating a general correlation of high CAIX intensity linked to high PC1 scores

In order to further correlate glycogen as measured through RS/PCA and CAIX IF, a pixel-by-pixel comparison between Raman maps and CAIX IF maps of the same tissue section was generated (Fig. 6). A qualitative comparison of PC1 and CAIX IF maps for both the 0 Gy (Fig. 6a, b) and 15 Gy (Fig. 6c, d) sections shows similarities in the cellular co-expression of glycogen and CAIX. A quantitative pixel-based comparison of the two images shows a general correlation between increased CAIX intensity and increased PC1 score within a 100 × 100 μm^2^ region. This result indicates that NSCLC tumour regions containing positive CAIX pixels spatially correlate to pixels with high PC1 scores and thus high glycogen content.

## Discussion

This study used RS to identify key metabolic changes in irradiated NSCLC tumour xenografts following RT. We focused on in vivo changes that resulted from radiation using H460 NSCLC tumour cells to corroborate our previous in vitro studies [36]. Changes in metabolic profile as indicated through PC1 were observed in this study, and corroborate previous work by our group in which radiation-induced metabolic alterations were detected in human NSCLC tumour xenografts through changes in glycogen content at 3 days post treatment [36]. Similarly, RS analysis in this study revealed in vivo glycogen levels differ between irradiated and unirradiated tumours as early as 2 h and as late as 10 days post treatment (Fig. 1c). By tracking glycogen metabolism and tumour area, the radiation-induced glycogen signature was found to be linearly correlated to tumour regression. Untreated tumours continued to grow and contained less glycogen than the irradiated counterparts. The latter tumours had slower growth rates or regressed and contained relatively more glycogen. One possible explanation for this is that larger tumours have an increased metabolic demand (cells are actively proliferating) and therefore consume more energy reserves by depleting cellular glycogen stores. Alternatively, exposure to radiation and concomitant increase in cellular stress may switch cells to preserve energy stores in the form of glycogen.

At 2 h post treatment we found reduced expression of the glycogen RS signature in irradiated tumours. We also show increased levels of γ-H2AX staining at 15 Gy, confirming radiation-induced DNA damage has occurred in the tumours (Fig. 3, Fig. 4a). One possibility is that within 2 h sufficient time has elapsed for the initial DNA damage response to have initiated and activated radiation-induced cell death [49–51]. Therefore an increase in PIMO positive staining in irradiated tumours could be an indication of selective cell kill towards oxygenated tumour regions at this time (Fig. 3, Fig. 4b). Taken together, reduced expression of the RS glycogen signature in irradiated tumours can be attributed to increased initial radiation-induced damage response within the tumour or possibly hypoxic tumours.

Unirradiated tumours harvested at 3 and 10 days showed increases in size and levels of hypoxia, which is consistent with increased demand for metabolic resources. In unirradiated tumours, an observed increase in hypoxia was associated with higher levels of γ-H2AX staining when compared to irradiated tumours that showed lower PIMO staining. This observation is corroborated in the literature [48]. These tumours also showed relatively lower glycogen RS scores compared to irradiated tumours, suggesting that cells may have adapted to high energy metabolic demand from hypoxic damage.

While hypoxic cells are typically associated with upregulation of glycogen synthesis and thus increased glycogen levels [52], RS identifies relative glycogen levels. In this case, hypoxic, unirradiated tumours have relatively lower glycogen levels compared to the less hypoxic irradiated tumours. While this may seem counter-intuitive that unirradiated tumours display lower glycogen yet a higher extent of tumour hypoxia, it is important to note that the measured changes observed in this reported study are the relative glycogen differences between irradiated/unirradiated. This implies that radiation-induced glycogen increases may be greater than the effect of the regulation of glycogen by hypoxia. Indeed, unirradiated tumours exhibit detectable levels of glycogen, even at day 3 (Fig. 1c).

The presence of glycogen in unirradiated tumours has been previously confirmed using a semi-quantitative colorimetric assay by our group [36].

In contrast to unirradiated controls, irradiated tumours harvested at 3 days post treatment contained relatively higher glycogen levels, a time point where repaired cells are recovering their bioenergetics. These tumours were found to be smaller compared to the unirradiated controls, but also had less DNA damage and hypoxia. While there were no direct measurements of tumour reoxygenation, we inferred the relative extent of hypoxia and reoxygenation by coupling CAIX and PIMO staining. CAIX is a relatively stable transmembrane protein and has been shown to remain present in cells even during recovery from hypoxic conditions, with significant positive staining shown up to 48 h into reoxygenation for some cell lines [53]. On the other hand, PIMO positivity captures earlier hypoxia events whereby PIMO is directly taken up in oxygen poor cells. A reduction in PIMO positive staining and greater CAIX staining in irradiated tumours compared to unirradiated tumours could indicate an oxygen recovering state. In addition to this, irradiated tumours decreased in area at 3 days post treatment. As a consequence, fewer cells are available to deplete tumour resources compared to before treatment. This in turn may lead to improved tumour reoxygenation. Furthermore, hypoxic tissue is more radioresistant than normoxic tissue, therefore it is likely that a large portion of the tissue remaining in irradiated tumours at 3 days post irradiation was hypoxic at time of treatment but became reoxygenated at 3 days post-irradiation. Therefore the concurrent decrease in PIMO staining and relatively higher CAIX staining 3 days following irradiation may support the notion that irradiated tumours are recovering from hypoxia. The glycogen rich irradiated tumours appear to contain a higher concentration of cells that were hypoxic at the time of treatment and are reoxygenated at 3 days post treatment, compared to unirradiated controls.

Our results suggest that RS is able to not only identify glycogen signatures but track these changes over a defined period of 10 days. Importantly, the changes in glycogen were strongly associated with response of the tumour as a result of radiation-induced DNA damage and tissue oxygenation. Specifically, we show that relatively lower levels of cellular glycogen are associated with larger tumours that have increased levels of hypoxia and DNA damage in the tumour. In contrast, higher glycogen levels are associated with relatively smaller tumours that have less hypoxia and minimal DNA damage, or treated tumours that have become reoxygenated.

The NOD/SCID mice used in this study are devoid of both their T and B-cell compartments, therefore, the contribution of the immune system to the overall response to radiation treatment is not considered. The dominant Raman spectral signatures observed in the current model could potentially differ from what would be observed in the case of an intact immune system. However by knowingly tracking a glycogen signature in the Raman spectrum, NSCLC tumour oxygenation trends can still be monitored.

Previously, the human breast MDA-MB-231 tumour model was also studied using RS. In this case, RS identified a radiation response signature linked to changes in specific proteins (including tryptophan, proline, phenylalanine and β-sheet amide bands), and no involvement of glycogen related Raman bands was observed [37]. Therefore, to date, the NSCLC H460 tumour model is the only in vivo model for which the radiation-induced glycogen Raman signature has been recovered. This suggests radiation-related Raman signatures are tumour-specific, varying based on both tumour origin and strain. Future studies will be required with a broader examination of other tumour cell lines and their responses in vivo to validate the radiation induced responses seen here. Indeed, the predominant biochemical changes occurring within a tumour in response to irradiation or hypoxia that are observed using RS are not necessarily expected to be the same from one sub-type to the next, further emphasizing the need for personalized tracking techniques such as RS.

## Conclusion

Raman spectroscopy offers a label-free way to monitor NSCLC response to radiation by measuring metabolic alterations within the tumour as a result of oxygenation state of the tissue. We show that RS can be used as early as 2 h post irradiation to measure acute metabolic signs of the NSCLC radiation response, which is associated with elevated levels of hypoxia in the tumour. We also show that a radiation-induced increase in glycogen can be detected as early as 1 day and up to 10 days post irradiation using RS, and is associated with changes that are phenotypic of tissue reoxygenation. This study demonstrates the use of RS in detecting metabolic changes that are associated with radioresistance of NSCLC and opens up new pathways that could potentially exploited to radiosensitize tumours.

## Additional file

Additional file 1: **Table S1.** Raman peak assignment for radiation induced principal component 1 (PC1). **Table S2.** Raman peak assignment for principal component 2 (PC2). **Figure S1.** Mean Raman spectrum obtained for unirradiated and irradiated tumours harvested at 2 hours and 3 days post treatment. **Figure S2.** PC2 score in relation to tumour regression. **Figure S3.** No correlation between PC2 score and CAIX IF intensity. (ZIP 2231 kb)

## Abbreviations

BSA: Bovine Serum Albumin Fraction V
CAIX: Carbonic anhydrase IX
CBCT: Cone beam computed tomography
CCD: Charge coupled device
HIF-1: Hypoxia inducible factor 1
IF: Immunofluorescence
NSCLC: Non-small cell lung carcinoma
PBS: Phosphate buffer saline
PC: Principal component
PCA: Principal component analysis
PIMO: Pimonidazole
RS: Raman spectroscopy
RT: Radiation therapy
SARRP: Small animal radiation research platform
SBRT: Stereotactic body radiation therapy
γ-H2AX: Phospho-histone H2AX
