# Supplementary material for: Raman spectroscopy detects metabolic signatures of radiation response and hypoxic fluctuations in non-small cell lung cancer
Source: BMC Cancer. 2019 May 20;19:474. doi: 10.1186/s12885-019-5686-1 (PMC6528330; doi:10.1186/s12885-019-5686-1)
Supplement: Supplementary file 1 — Table S1. Raman peak assignment for radiation induced principal component 1 (PC1). Table S2. Raman peak assignment for principal component 2 (PC2). Figure S1. Mean Raman spectrum obtained for unirradiated and irradiated tumours harvested at 2 hours and 3 days post treatment. Figure S2. PC2 score in relation to tumour regression. Figure S3. No correlation between PC2 score and CAIX IF intensity. (ZIP 2231 kb) [file 12885_2019_5686_MOESM1_ESM.zip › Suppl_FigureS1R3.pdf]

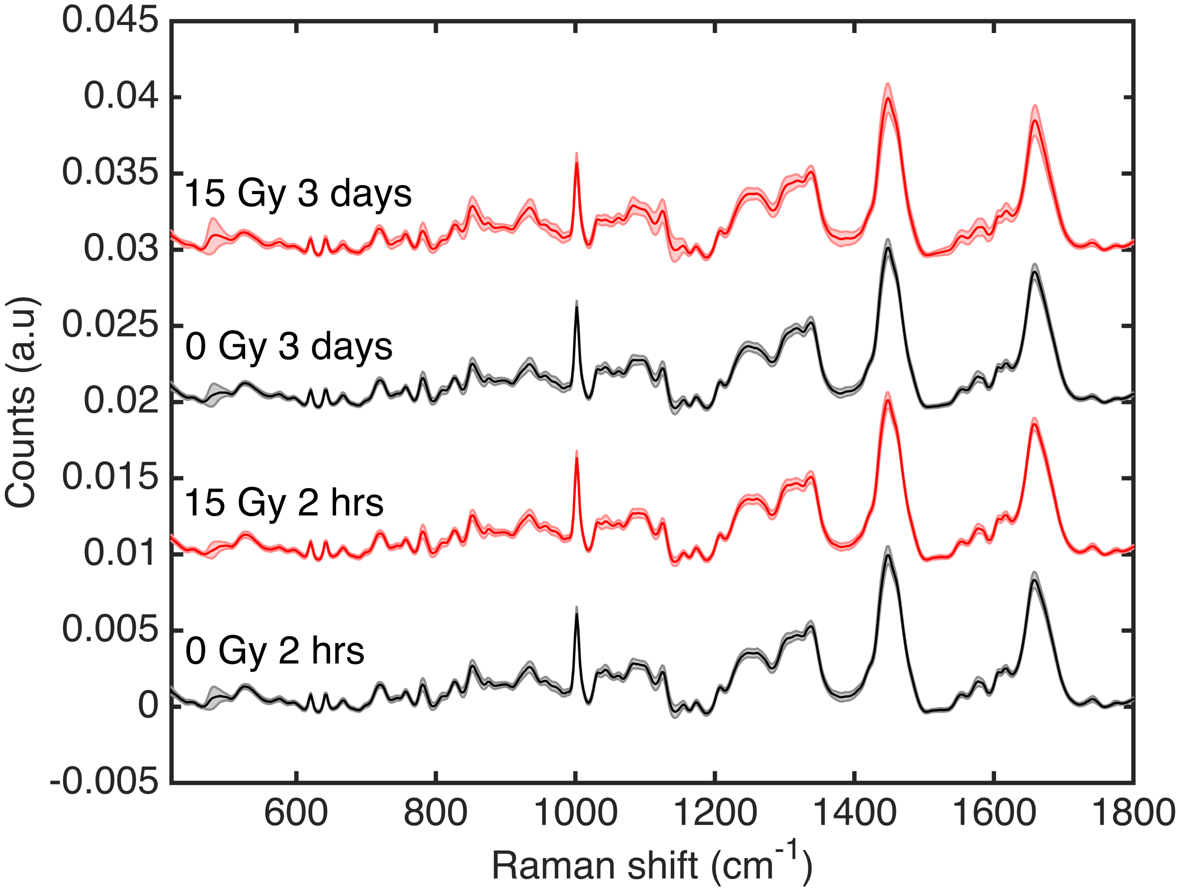

Figure S.1 Mean Raman spectrum obtained for unirradiated and irradiated tumours harvested at 2 hours and 3 days post treatment. Mean Raman spectrum (solid trace) is plotted for H460 tumours and standard deviation (+/-) is shown (shading) to illustrate the variation in Raman bands present in the spectra making up each dose group.
