# Supplementary material for: Raman spectroscopy detects metabolic signatures of radiation response and hypoxic fluctuations in non-small cell lung cancer
Source: BMC Cancer. 2019 May 20;19:474. doi: 10.1186/s12885-019-5686-1 (PMC6528330; doi:10.1186/s12885-019-5686-1)
Supplement: Supplementary file 1 — Table S1. Raman peak assignment for radiation induced principal component 1 (PC1). Table S2. Raman peak assignment for principal component 2 (PC2). Figure S1. Mean Raman spectrum obtained for unirradiated and irradiated tumours harvested at 2 hours and 3 days post treatment. Figure S2. PC2 score in relation to tumour regression. Figure S3. No correlation between PC2 score and CAIX IF intensity. (ZIP 2231 kb) [file 12885_2019_5686_MOESM1_ESM.zip › Suppl_TableS1R3.pdf]

Supplementary Table S.1: Raman peak assignment for radiation induced principal component 1 (PC1). Only main spectral contributors to the PC are listed.

	Assignment			
**Raman Shift (cm^{-1})**	**Nucleic Acid**	**Protein**	**Lipid**	**Carbohydrate**
479				glyc.
580				glyc.
726	A[1]			
784	C,U ring str. [2]			
810			O-P-O[3]	
850				glyc.
940				glyc.
955-994	d.[4]	C-C str.[4]	C-C[3]	
1002		phe. sym. rb.[3]		
1042				glyc.
1082				glyc.
1129				glyc.
1178		phe. ring str.[5]		
		tyr.[6]		
1249	T[4]	am. III β[3]		
1297	A[7]	CH def. [7]	CH_2 tw.[3]	
1335				glyc.
1382				glyc.

	Assignment			
**Raman Shift (cm^{-1})**	**Nucleic Acid**	**Protein**	**Lipid**	**Carbohydrate**
1442		CH def.[3]	CH def.[3]	
1659	T, C, G[4]	am. I[4]		

Glycogen (glyc), tyrosine (tyr), adenine (A), cytosine (C), uracil (U), stretch (str), deoxyribose (d), phenylalanine (phe), symmetric (sym), ring breathing (rb), vibration (vib), bending (bd), thymine (T), amide (am), β sheet (β), deformation (def), twist (tw), guanine (G).

References

[1] Krafft C, Knetschke T, Siegner A, Funk RHW, Salzer R. Mapping of single cells by near infrared Raman microspectroscopy. Vib Spectrosc 2003;32:75–83. doi:10.1016/S0924-2031(03)00049-3.

[2] Naumann D. FT-infrared and Raman spectroscopy in biomedical research. Appl Spectrosc Rev 2001;36:239–98.

[3] Notingher I, Verrier S, Haque S, Polak JM, Hench LL. Spectroscopic study of human lung epithelial cells (A549) in culture: Living cells versus dead cells. Biopolym - Biospectroscopy Sect 2003;72:230–40. doi:10.1002/bip.10378.

[4] Overman SA, Aubrey KL, Reilly KE, Osman O, Hayes SJ, Serwer P, et al. Conformation and interactions of the packaged double-stranded DNA genome of bacteriophage T7. Biospectroscopy 1998;4:S47–56. doi:10.1002/(SICI)1520-6343(1998)4:5+<S47::AID-BSPY6>3.0.CO;2-7.

[5] Omberg KM, Osborn JC, Zhang SL, Freyer JP, Mourant JR, Schoonover JR. Raman spectroscopy and factor analysis of tumorigenic and non-tumorigenic cells. Appl Spectrosc 2002;56:813–9. doi:10.1366/000370202760171464.

[6] Uzunbajakava N, Lenferink a., Kraan Y, Willekens B, Vrensen G, Greve J, et al. Nonresonant Raman imaging of protein distribution in single human cells. Biopolym - Biospectroscopy Sect 2003;72:1–9. doi:10.1002/bip.10246.

[7] Puppels GJ, Garritsen HS, Segers-Nolten GM, de Mul FF, Greve J. Raman microspectroscopic approach to the study of human granulocytes. Biophys J 1991;60:1046–56. doi:10.1016/S0006-3495(91)82142-7.
