# Supplementary material for: Raman spectroscopy detects metabolic signatures of radiation response and hypoxic fluctuations in non-small cell lung cancer
Source: BMC Cancer. 2019 May 20;19:474. doi: 10.1186/s12885-019-5686-1 (PMC6528330; doi:10.1186/s12885-019-5686-1)
Supplement: Supplementary file 1 — Table S1. Raman peak assignment for radiation induced principal component 1 (PC1). Table S2. Raman peak assignment for principal component 2 (PC2). Figure S1. Mean Raman spectrum obtained for unirradiated and irradiated tumours harvested at 2 hours and 3 days post treatment. Figure S2. PC2 score in relation to tumour regression. Figure S3. No correlation between PC2 score and CAIX IF intensity. (ZIP 2231 kb) [file 12885_2019_5686_MOESM1_ESM.zip › Suppl_TableS2R3.pdf]

Supplementary Table S.2: Raman peak assignment for principal component 2 (PC2). Only main spectral contributors to the PC are listed.

	Assignment			
**Raman Shift (cm^{-1})**	**Nucleic Acid**	**Protein**	**Lipid**	**Carbohydrate**
666	G, T[1,2]			
714			cho.[1]	
			C-N str.[3]	
726	A[1]			
784	C, U[3]			
855	O-P-O str.[4]	tyr. rb.[1]		
927		C-C bk. α[5]		
1004		phe. sym. rb.[1]		
1071		C-N str.[4]	C-C str.[1]	
1134		C-N str.[4]	C-C str.[1,4]	
1178	DNA/RNA ring vib.[6]	phe. ring str.[6]		
		C-H bd. tyr.[4,6]		
1235		am III rc.[4]		
1272		am III α[4,5]		
1297		am III α[4]	CH def.[4]	
1338	CH_3CH_2 wg.[3]			
1373	T[1]			
	A, G[2]			

	Assignment			
**Raman Shift (cm^{-1})**	**Nucleic Acid**	**Protein**	**Lipid**	**Carbohydrate**
1438			CH_2 def.[1]	
1577	A, G[1]			
1618		tyr.[7]		
		trp.[3]		
1659		am. I α[3]	C=C str.[3]	
1682		am. I β[5]		

Guanine (G), thymine (T), choline (cho), stretch (str), adenine (A), cytosine (C), uracil (U), tyrosine (tyr), ring breathing (r.b.), backbone (bk.), α helix (α), phenylalanine (phe), symmetric (sym), vibration (vib), bending (bd), wagging (wg), deformation (def), tryptophan (trp), amide (am), β sheet (β), random coil (rc).

References

[1] Krafft C, Knetschke T, Siegner A, Funk RHW, Salzer R. Mapping of single cells by near infrared Raman microspectroscopy. Vib Spectrosc 2003;32:75–83. doi:10.1016/S0924-2031(03)00049-3.

[2] Overman SA, Aubrey KL, Reilly KE, Osman O, Hayes SJ, Serwer P, et al. Conformation and interactions of the packaged double-stranded DNA genome of bacteriophage T7. Biospectroscopy 1998;4:S47–56. doi:10.1002/(SICI)1520-6343(1998)4:5+<S47::AID-BSPY6>3.0.CO;2-7.

[3] Stone N, Kendall C, Shepherd N, Crow P, Barr H. Near-infrared Raman spectroscopy for the classification of epithelial pre-cancers and cancers. J Raman Spectrosc 2002;33:564–73. doi:10.1002/jrs.882.

[4] Notingher I, Verrier S, Haque S, Polak JM, Hench LL. Spectroscopic study of human lung epithelial cells (A549) in culture: Living cells versus dead cells. Biopolym - Biospectroscopy Sect 2003;72:230–40. doi:10.1002/bip.10378.

[5] Synytsya A, Alexa P, Besserer J, De Boer J, Froschauer S, Gerlach R, et al. Raman spectroscopy of tissue samples irradiated by protons. Int J Radiat Biol 2004;80:581–91. doi:10.1080/09553000412331283515.

[6] Omberg KM, Osborn JC, Zhang SL, Freyer JP, Mourant JR, Schoonover JR. Raman spectroscopy and factor analysis of tumorigenic and non-tumorigenic cells. Appl Spectrosc 2002;56:813–9. doi:10.1366/000370202760171464.

[7] Naumann D. FT-infrared and Raman spectroscopy in biomedical research. Appl Spectrosc Rev 2001;36:239–98.
